# Progress in the application of molecular imaging technology in immunological tolerance and immune metabolism visualization research

**DOI:** 10.3389/fimmu.2025.1583228

**Published:** 2025-04-01

**Authors:** Kailang Li, Fang Xie, Yongfu Xiong, Jin Jiang, Bifan Huang

**Affiliations:** ^1^ Department of Radiology, The Third People’s Hospital In Xindu District of Chengdu, Chengdu, China; ^2^ Department of Oncology, The Second People’s Hospital of Yibin, Yibin, China; ^3^ Department of General Surgery, Affiliated Hospital of North Sichuan Medical College, Nanchong, China; ^4^ Department of Radiology, Sichuan Provincial People’s Hospital, University of Electronic Science and Technology of China, Chengdu, China

**Keywords:** molecular imaging, immune tolerance, immune metabolism, immunotherapy, visualization technology

## Abstract

Immunological tolerance and immune metabolism play crucial roles in maintaining immune homeostasis and the immune response to diseases. The advancement of molecular imaging technologies, particularly optical molecular imaging, nuclear medicine imaging, and magnetic resonance imaging, has led to a significant progress in the visualization of immune tolerance and immune metabolism. Molecular imaging technologies enable real-time monitoring and analysis of dynamic changes in immune tolerance mechanisms and immune metabolism in living organisms, allowing the development of new strategies for early disease diagnosis, targeted therapy, and immunotherapy. This article reviews the latest advancements in the application of molecular imaging technologies in the fields of immunological tolerance and immune metabolism, with a focus on their applications in the regulation of immune tolerance regulation, immune metabolism, and immunotherapy.

## 1 Introduction

Immunological tolerance refers to the state in which the immune system does not respond to self-antigens, preventing the body from attacking its own tissues, which is crucial to avoid developing autoimmune diseases (1). Immune metabolism, on the other hand, refers to the processes by which immune cells regulate their functions through changes in metabolic pathways during immune responses (2). In recent years, both immunological tolerance and immune metabolism have become hot topics in immunology research, attracting considerable academic attention. However, traditional research methods often rely on *in vitro* analyses or examination of tissue sections, which do not allow to monitor real-time dynamic processes in living organisms.

**Figure 1:**
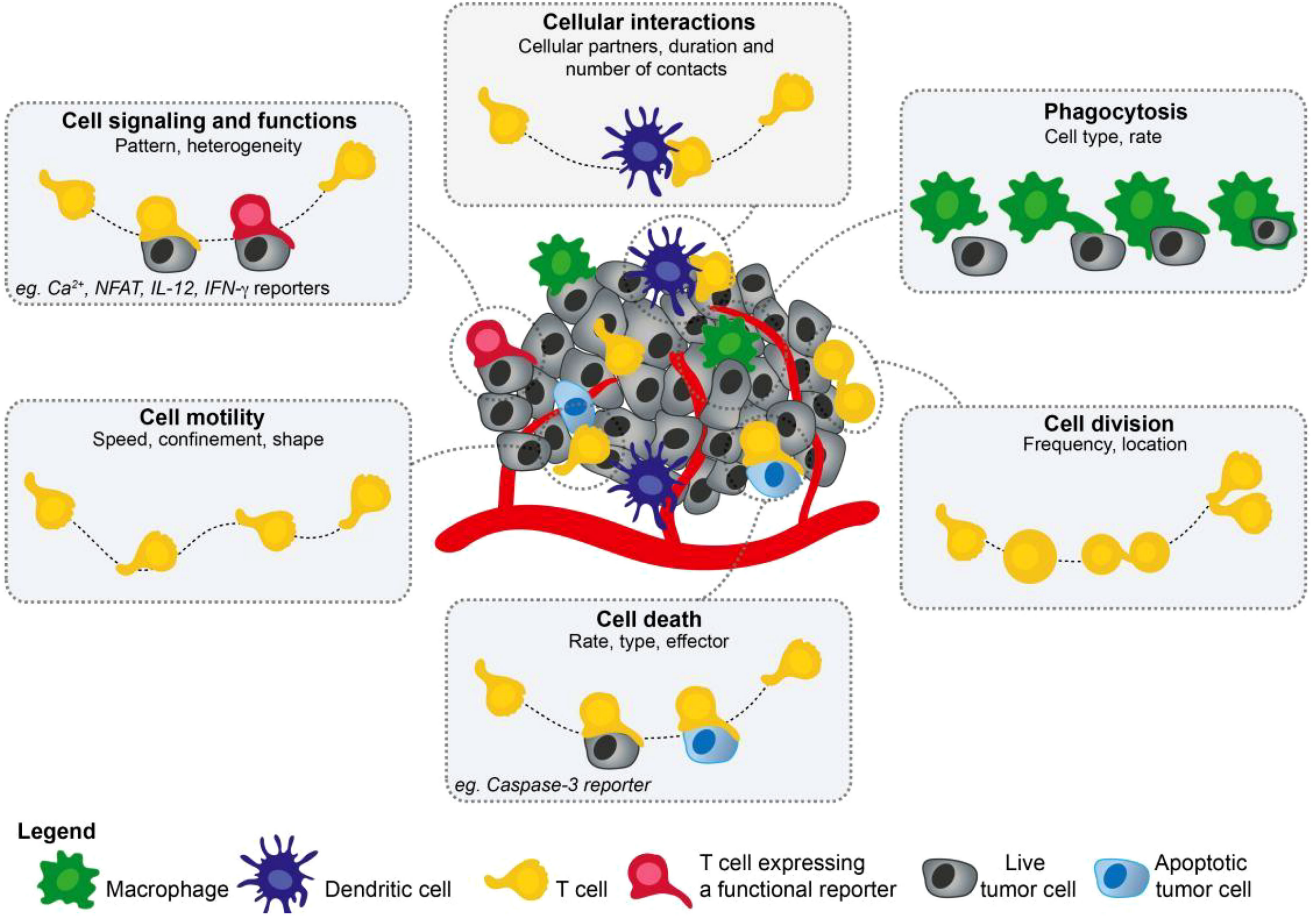
This figure illustrates the diversity of parameters that can be collected using two-photon microscopy. Cell migration, cellular interactions, cell division, or phagocytosis are readily visualized using fluorescently labeled cells but the introduction of functional reporters has extended the list of measurable parameters, including cell signaling, cell death, or gene expression (5). Adapted with permission from copyright 2019, Elsevier Ltd.

With the recent advancements in imaging technologies and development of new tracers, molecular imaging has shown great potential in revealing the dynamic processes and metabolic activities of the immune system. As a high-resolution imaging technique capable of real-time monitoring, quantitative analysis, and spatial localization in living organisms, molecular imaging is an ideal tool for studying the dynamic processes of immune tolerance and immune metabolism (3, 4). Various molecular imaging techniques can monitor biological processes, such as immune cell migration, proliferation, and metabolic state changes, by labeling specific molecular targets or metabolic products (**Figure 1** **) (** 5). For example, molecular imaging can be used to monitor the localization and function of immune cells in cancer, which is critical for determining the effectiveness of immunotherapy (6). Therefore, the use of molecular imaging technology shows a great potential in immunology research, particularly for monitoring immune tolerance and immune metabolism.

This review aims to examine the progress in the application of molecular imaging techniques in monitoring immune tolerance and immune metabolism, providing new research ideas and technical support for researchers in related fields.

## 2 Overview of molecular imaging technology

Molecular imaging is a broad field that encompasses various imaging techniques, and thus the concept of molecular imaging can be understood as a variety of imaging techniques that can reveal and differentiate various physiological and pathological processes at the cellular or subcellular level in living organisms (7). In recent years, molecular imaging technologies have developed rapidly, particularly in the field of immunology, and are expected to make significant contributions to advancing our understanding of immune mechanisms, diagnostics, and therapies.

First, molecular imaging techniques are used to evaluate changes at the cellular level, which occur much earlier than changes at the anatomical level of the organism, thus providing sensitive tools for early monitoring of immune tolerance or immune metabolism. Additionally, these techniques detect functional changes within tissues rather than structural changes, which enhances their sensitivity (8). Another notable application of molecular imaging is in the preclinical evaluation of new drugs, particularly immunotherapies, where it allows researchers to quantitatively monitor the effects of drugs on molecular targets within living animals.

Certainly, molecular imaging is a very broad field, and there are a variety of methods available to perform molecular imaging. Commonly used molecular imaging techniques include optical imaging, nuclear medicine imaging, magnetic resonance imaging (MRI), and ultrasound imaging, *etc* (9). In the field of immune tolerance and immune metabolism research, the most commonly used molecular imaging techniques include the following:

### 2.1 Optical molecular imaging

Optical molecular imaging techniques can detect light signals emitted by endogenous or exogenous agents *in vivo* or in tissues, revealing the encoded information from microscopic biochemical processes in the body to the observer. Optical molecular imaging techniques, with bioluminescent or fluorescent markers, provide high spatial resolution real-time imaging, making them particularly suitable for small animal model research. For instance, Yoon et al. developed the near-infrared fluorophore ESNF13 and used optical imaging technology to track the biodistribution and accumulation patterns of natural killer (NK) cells in the tumor sites of a triple-negative breast cancer xenograft mouse model in real-time (10).

### 2.2 Nuclear medicine imaging

Nuclear medicine imaging techniques use radiolabeled compounds to obtain images that reveal information about organ/tissue function and metabolism, offering high sensitivity and the advantage of quantitative analysis, which is often used to detect metabolic activity in cells and tissues. Techniques like positron emission tomography (PET) and single-photon emission computed tomography (SPECT) enable non-invasive monitoring of physiological and pathological processes in the body using radioactive tracers. These techniques can detect pathological changes at the early stages of disease, enabling earlier disease recognition than traditional anatomical-based imaging techniques (11). Recently, nuclear medicine imaging is increasingly being used in immunotherapy for response assessment. The emergence of immune checkpoint inhibitors and other immunotherapies has dramatically changed cancer treatment. Nuclear medicine imaging can help evaluate the response to immunotherapy, assess immune-related adverse effects, and predict treatment efficacy based on imaging features (12).

### 2.3 Magnetic resonance imaging

In recent years, MRI has been used to examine and track key events in inflammatory processes, which has significant implications for disease monitoring and drug development. MRI enables real-time imaging of dynamic changes in immune cells in the body, providing new insights for understanding the pathophysiology of diseases (13). Additionally, significant progress has been made in MRI cell tracking to monitor immune cells. For example, non-invasive tracking of macrophages can be achieved by labeling these cells with superparamagnetic iron oxide nanoparticles. This method not only enhances MRI contrast but also allows real-time monitoring of immune cell migration and distribution *in vivo*, providing a powerful tool for studying immune responses (14). More importantly, the advent of dynamic nuclear polarization-enhanced magnetic resonance imaging (DNP-EMRI) has allowed the observation and monitoring of metabolic processes *in vivo*. This technique enhances the signal strength of metabolites, enabling detailed studies of metabolic characteristics in diseases such as cancer. DNP-EMRI can track dynamic changes in metabolites in real-time, providing new methods for the diagnosis and treatment of cancer (15).

## 3 Application of molecular imaging techniques in immune tolerance research

The development of immune tolerance involves a complex interplay among multiple immune cell types, including dendritic cells, regulatory T cells (Tregs), and other cell populations of the immune system. Molecular imaging techniques have played an important role in revealing the dynamic processes of immune tolerance.

### 3.1 Role of dendritic cells in immune tolerance

Dendritic cells are important immune system cells, which act as immune sensors coordinating immune reactions that can initiate or suppress immune responses by capturing and presenting antigens. During the process of immune tolerance, dendritic cells play a key regulatory role (16). Molecular imaging techniques have been widely used to track dendritic cells *in vivo*. By using fluorescently or radioactively labeled dendritic cells, researchers can monitor in real-time the activation and migration of dendritic cells in different immune environments (17). For example, using optical molecular imaging technology and labeled dendritic cells in a mouse model, studies have found significant changes in dendritic cell migration patterns during the process of immune tolerance. Under different immune tolerance conditions, the activity and metabolic state of dendritic cells also showed significant changes, providing further insights for deeper understanding of the mechanisms of immune tolerance (18).

### 3.2 Role of regulatory T cells in immune tolerance

Tregs play a crucial role in maintaining immune tolerance by suppressing autoimmune responses through multiple mechanisms, thereby preventing the immune system from attacking the body’s own tissues. Molecular imaging techniques can be used to monitor the dynamic changes of Tregs in immune tolerance, especially in the context of tumor immune tolerance.

By using fluorescently or radioactively labeled Tregs, researchers can monitor in real-time the localization and proliferation of Tregs during the development of immune tolerance (19). For example, nuclear medicine imaging techniques using radiolabeled Tregs can precisely detect changes in Treg activity under specific immune tolerance conditions, providing important information for the study of immune tolerance (20).

### 3.3 Imaging biomarkers for immune tolerance

In recent years, researchers have undertaken the development of imaging biomarkers for immune tolerance. These biomarkers can be used in real-time monitoring of the development and maintenance of immune tolerance. For instance, certain antibodies or small molecule probes have been used to specifically label regulatory immune cells or molecules related to immune tolerance (21). The application of these molecular imaging biomarkers in immune tolerance research offers new options for early disease diagnosis and the optimization of immunotherapies.

## 4 Application of molecular imaging techniques in immune metabolism research

Immune metabolism refers to the dynamic changes in metabolic pathways in immune cells during different immune responses (22). As the immune system responds to pathogens, cancer cells, and other pathological agents entering the body, the metabolic state of immune cells undergoes significant changes, and molecular imaging techniques allow us monitor these changes in real-time.

### 4.1 Regulation of immune cell metabolism

The metabolic state of immune cells directly affects their function and the strength of immune responses mediated by them. In recent years, molecular imaging techniques have been used to investigate the regulatory mechanisms of immune cell metabolism. For example, non-invasive molecular imaging techniques like PET and MRI have been used to study postprandial energy metabolism in humans, particularly the metabolism of glucose and fatty acids during the post-meal state (23). Additionally, the development of fluorescent probes has provided new tools for real-time monitoring of intracellular metabolites. These fluorescent probes can be used to track key metabolites, such as adenosine triphosphate, cyclic adenosine monophosphate, and cyclic guanosine monophosphate, helping researchers better understand the role of metabolic pathways in cellular activities (24). The combination of these techniques not only provides important tools for basic research but also offers new options for clinical diagnosis and treatment.

In immune cells, changes in metabolic pathways are also of great importance. Immune cell activation requires metabolic reprogramming to support their proliferation, chemotaxis, and cytokine production. Studies have shown that the reprogramming of metabolic pathways has a direct impact on the regulation of immune cell functions, providing a new direction for immunometabolism research (25). By studying metabolic pathways in greater depth, we can gain a better understanding of the complex functions of the immune system in both healthy and pathological states.

### 4.2 Immune metabolism and tumor immunotherapy

Tumor immunotherapy is a key area of research in cancer treatment. The metabolic changes in the tumor immune microenvironment significantly impact the effectiveness of immunotherapy. Molecular imaging techniques have been used to monitor metabolic changes in immune cells during tumor immunotherapy, in order to evaluate treatment efficacy. For example, fluorodeoxyglucose (18F-FDG) has been used with PET imaging techniques to observe the metabolic activity of immune cells in the tumor immune microenvironment, providing guidance for personalized tumor immunotherapy strategies (26). Tumor cells can evade immune surveillance by altering the glucose and glutamine metabolic pathways, thereby affecting the expression of PD-L1, which in turn affects the efficacy of PD-1/PD-L1 inhibitors (27).

### 4.3 Immune metabolism and biomarkers

**Figure 2:**
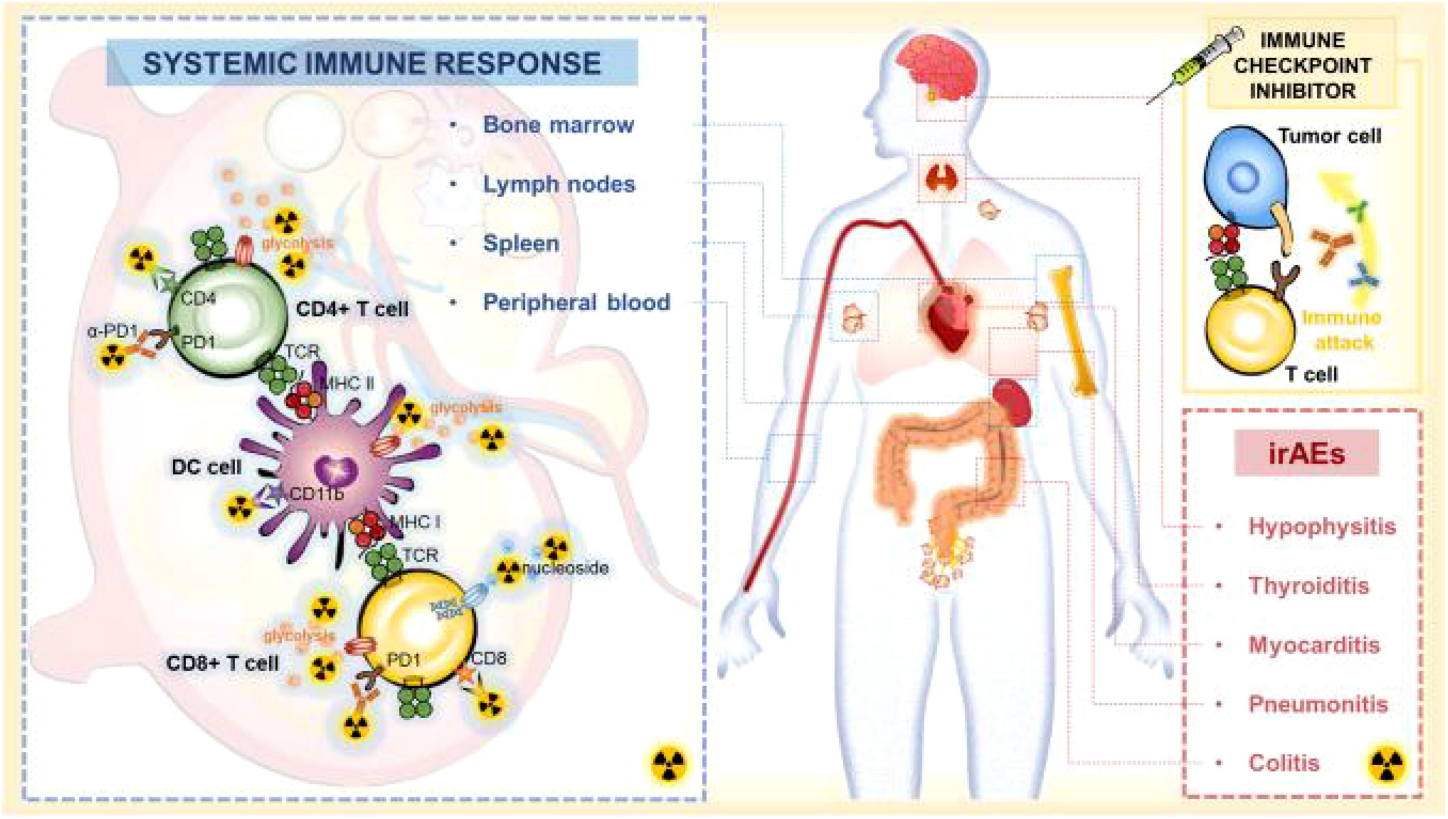
Systemic immune response to immune checkpoint inhibitors. When ICI treatment triggers effective immune activation, the complex changes including metabolic patterns and cellular dynamics occur in systemic immune cells. PET-based imaging can detect changes in metabolic markers or specific biomarkers of systemic immune responses to assess immune activation and predict irAEs after ICI therapy (28). Adapted with permission from copyright 2023, The Author(s).

In the last few years, significant progress has been made in the development of imaging biomarkers for monitoring immune metabolism. These biomarkers play an important role in the diagnosis and treatment of cancer, neuroinflammation, and other immune-related diseases. For example, PET molecular imaging technology has been widely used in cancer diagnosis, treatment evaluation, follow-up, and prognosis prediction. PET imaging can monitor metabolic changes in immune cells and specifically detect immune biomarkers, indicating systemic immune responses (**Figure 2**) (28). Additionally, in neuroinflammation research, imaging techniques such as near-infrared spectroscopy, transcranial Doppler imaging, elastography, electroencephalography, MRI, spectral analysis, and cytokine analysis techniques have been used to measure cell metabolism biomarkers related to neuroinflammation. These techniques have the potential to provide real-time information and guide more informed therapeutic decisions (29).

In the field of immune metabolism, research has also highlighted the importance of metabolic reprogramming in regulating immune cell functions. For example, immune metabolic reprogramming in microglial cells and its impact on neuronal activity have been revealed by fluorescence lifetime imaging microscopy. Studies have also found that microglial cells undergo metabolic rearrangement upon immune stimulation, and this change is crucial for controlling immune functions (30).

## 5 Application of molecular imaging techniques in immunotherapy

Molecular imaging techniques play an increasingly important role in cancer immunotherapy. Through non-invasive methods, molecular imaging techniques can be used to visualize and measure biological processes *in vivo*, providing crucial information for cancer diagnosis, treatment choices, and treatment efficacy assessment. For example, techniques such as PET and SPECT can be used to determine the biodistribution, expression, and heterogeneity of tumor antigens, helping with disease diagnosis, treatment selection, and patient stratification (31).

In recent years, immunotherapy, especially immune checkpoint inhibitors, has shown significant effects in the treatment of various cancers (32). However, due to the unique mechanisms of immunotherapy, traditional imaging techniques face challenges in assessing tumor response and progression. Molecular imaging technology can offer more precise evaluation techniques by visualizing specific target molecules in immune checkpoint pathways (33). Additionally, molecular imaging technology can be used to monitor immune-related adverse events induced by immunotherapy, thereby enhancing the safety and effectiveness of treatment (12).

In clinical applications, molecular imaging techniques, such as immune PET imaging, have demonstrated success in diagnosing and staging cancer. Through the use of radiolabeled antibodies and antibody fragments, immune PET imaging can quantitatively image biomarkers on the surface of cancer cells, providing a non-invasive approach for personalized treatment (34). Moreover, molecular imaging technology can be used to assess the efficacy of immunotherapy, helping identify biomarkers of early treatment response, thus optimizing treatment strategies (35).

The application of molecular imaging technology in immunotherapy extends beyond diagnosis and efficacy evaluation; it can also be used to guide treatment. For example, image-guided local immunotherapy can directly deliver immunotherapeutic agents to tumor sites, thereby improving the bioavailability of the treatment and reducing systemic toxicity (36). This approach combines imaging technology with therapeutic strategies, providing new options for cancer treatment. In conclusion, the prospects for molecular imaging technology in cancer immunotherapy are vast. With current advances in imaging technology and the development of new imaging probes, molecular imaging techniques will play a greater role in cancer immunotherapy, driving the development of personalized treatments.

## 6 Conclusion and outlook

Molecular imaging technology has played and continues to play an important role in the study of immune tolerance and immune metabolism, providing us with new approaches and tools. With the ongoing development of imaging technologies, particularly the optimization of molecular probes and imaging devices, we anticipate achieving more precise and real-time monitoring of immune tolerance and immune metabolism in the future.

However, the application of molecular imaging technology in immunological research still faces some challenges, including the need for improving image resolution, specificity, sensitivity of markers, and depth of tissue penetration, as well as overcoming other technical hurdles. Future research must address these technical bottlenecks to facilitate the broader application of molecular imaging technology in the field of immunology. For instance, by developing imaging equipment with better imaging performance, identifying target molecules with higher specificity, or developing immune imaging probes using the advantages of nanomedicine to promote the widespread application of molecular imaging techniques in the field of immunology.
